# Primary signet‐ring cell carcinoma of the renal pelvis: An autopsy case

**DOI:** 10.1002/iju5.12462

**Published:** 2022-05-11

**Authors:** Heisuke Iijima, Hiroaki Murata, Shinnosuke Oishi, Yusuke Kubota, Akihiko Sakamoto, Kuniaki Tanabe, Kazutaka Sugiyama, Akihiko Matsumoto, Ken Kuriki, Haruki Kume

**Affiliations:** ^1^ Department of Urology Yaizu City Hospital Yaizu Shizuoka Japan; ^2^ Department of Pathology Yaizu City Hospital Yaizu Shizuoka Japan; ^3^ Department of Urology The University of Tokyo Bunkyo‐ku Tokyo Japan

**Keywords:** autopsy, renal pelvic neoplasms, signet‐ring cell carcinoma, ureteral neoplasms, urologic neoplasms

## Abstract

**Introduction:**

Signet‐ring cell carcinoma is an extremely rare histological variant of upper urinary tract carcinoma, associated with poor prognosis.

**Case presentation:**

We report a case of a 75‐year‐old female diagnosed with left primary upper urinary tract signet‐ring cell carcinoma, initially treated with surgery. Post‐surgical development of multifocal metastases was followed by a devastating clinical course. An autopsy confirmed the tumor's primary origin in the upper urinary tract.

**Conclusion:**

We experienced a case of upper urinary tract signet‐ring cell carcinoma, with a rare opportunity to thoroughly confirm its primary site with an autopsy.

**Abbreviations & Acronyms**
5‐FU: 5‐fluorouracil
CA19‐9: cancer antigen 19‐9
CAT: scan‐Computed tomography scan
CDX2: caudal‐type homeobox transcription factor 2
CEA: carcinoembryonic antigen
CK20: cytokerain 20
CK7: cytokerain 7
GATA3: GATA binding protein 3
NSE: neuron‐specific enolase
SCC: squamous cell carcinoma
sIL‐2R: soluble interleukin 2 receptor
SRCC: signet‐ring cell carcinoma

**Keynote message**

A rare variant of upper urinary tract carcinoma was confirmed with post‐mortem examination.

## Introduction

Signet‐ring cell carcinoma in the urinary tract is an uncommon finding, and the bladder has been the primary site for the vast majority of these cases. Here, we report a rare case of upper urinary tract SRCC, confirmed by an autopsy.

## Case presentation

**Fig. 1:**
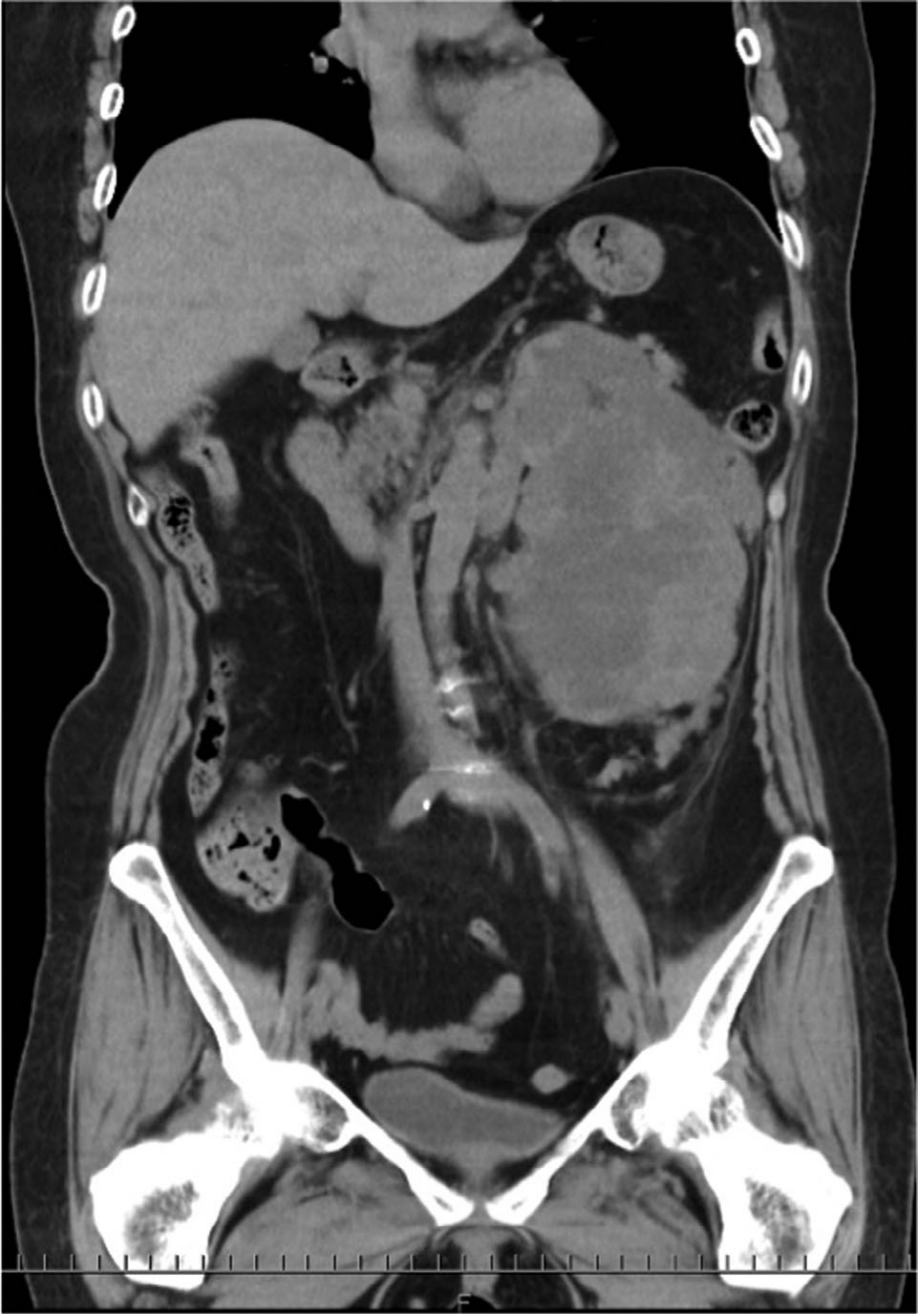
Computed tomography, coronal section showing left pelvic mass.

A 75‐year‐old previously healthy female was referred to our Urology Department for a large renal mass. She had noticed a palpable mass on her left flank half a year prior, which had been steadily increasing in size. She had also been experiencing dyspnea upon exertion in recent weeks. Her laboratory data showed elevated serum creatinine of 1.49 mg/dL, and mild hyperkalemia of 5.4 mEq/L. A non‐contrast CAT scan revealed a 16 × 10 cm mass almost replacing the left kidney and upper ureter, marked left hydronephrosis, and a paraaortic lymph node swelling of 22 mm, together suggesting malignant tumor of left renal or upper ureteral origin (Fig. 1). Serum tumor makers SCC, NSE, and sIL‐2R were slightly elevated, while CEA and CA19‐9 levels were normal, their values 1.7 ng/mL (normal range 0–1.5 ng/mL), 22.9 ng/mL (0–16.3 ng/mL), 630 U/mL (122–496 U/mL), 3.1 ng/mL (0–5.0 ng/mL), and 28.7 U/mL (0–37.0 U/mL), respectively.

**Fig. 2:**
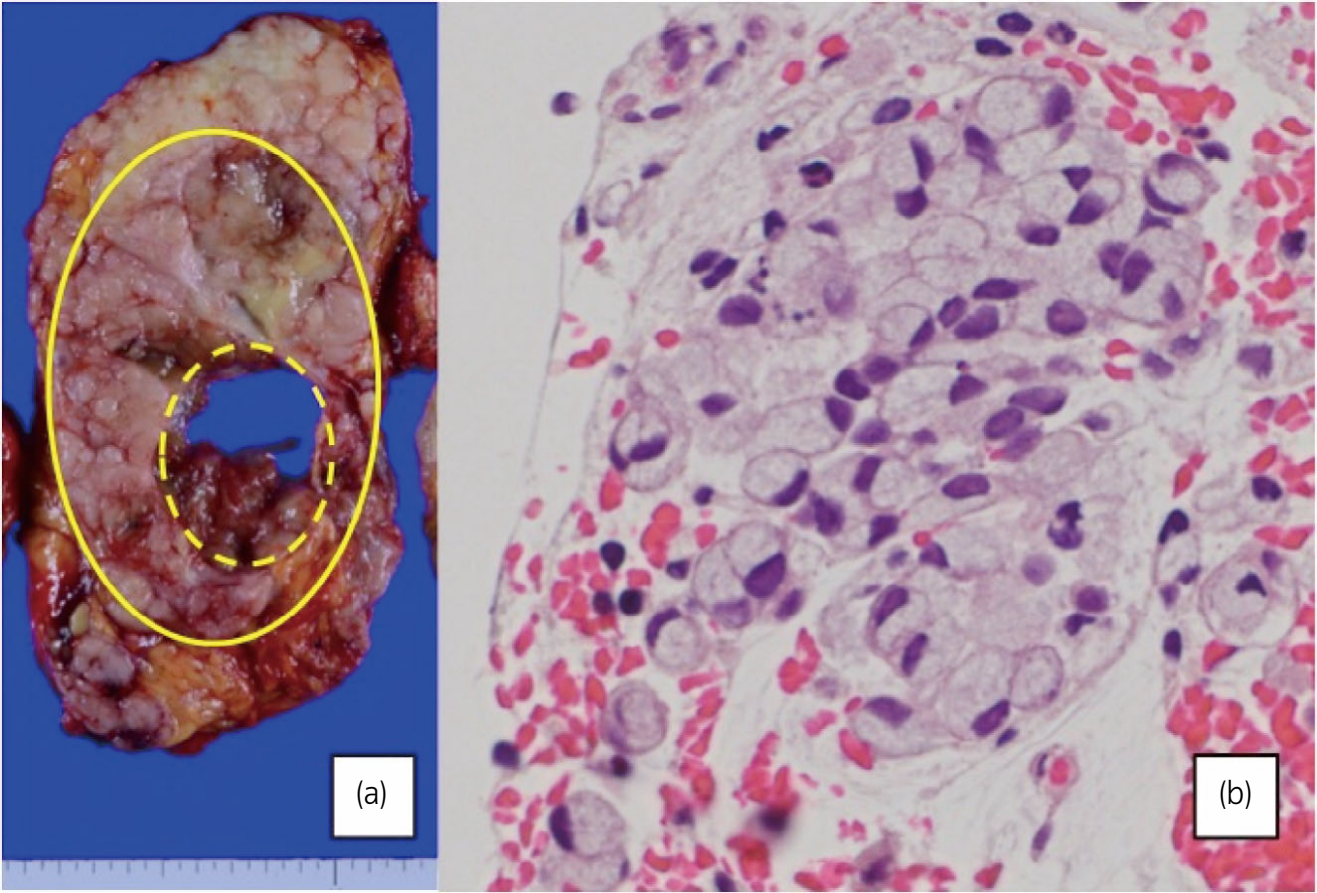
(a) Left kidney, post‐radical nephroureterectomy. Cut in coronal cross section. The renal pelvic tumor invades and completely replaces the kidney (circled in straight line). Hydronephrosis was also observed (dotted line). (b) H&E stain of the left renal pelvic tumor, revealing tumor cells with signet‐ring cell morphology. [Colour figure can be viewed at wileyonlinelibrary.com]

She underwent a retrograde pyelography and ureteroscopy, however, the guide‐wire and ureteroscope were unable to pass the ureteral obstruction at the U1‐U2 border. No visible lesions were observed on the ureteral mucosa up until the obstruction. Urine cytology sampled from this site was positive, and a cold‐cup biopsy revealed signet‐ring cell‐like cancer cells. A clinical staging of cT3N1M0 ureteral carcinoma was made, and she underwent an open radical left nephroureterectomy accompanied by paraaortic lymph node dissection 1 month after her initial hospital visit. The surgery was performed in the supine position, with Chevron incisions made for the nephrectomy, and Rutherford‐Morison incision for the ureterectomy. Most notably, strong adhesions were observed between the tumor and distal pancreas, as well as the spleen. The tumor was mucinous in appearance, and pathology determined a SRCC, originating from the renal pelvis (Fig. 2a,b). Lymph node metastases were also confirmed. The pathological stage was pT4N1, with positive surgical margins.

She was started on adjuvant Gemcitabine plus Carboplatin, however, chemotherapy was discontinued after the second course due to grade 2 erythema multiforme. A small papillary tumor was detected in the bladder 9 months after surgery, and transurethral resection of this bladder tumor was performed, confirming pTa urothelial carcinoma. Periodic CAT scans were negative for distant metastasis up until this point in her follow‐up.

One year after the initial surgery, metastases in the right lung, mediastinum, and sacrum were detected. Salvage chemotherapy with Pembrolizumab was scheduled, though her rapidly deteriorating overall health did not allow for its induction. She died 2 months after the initial confirmation of metastases.

**Fig. 3:**
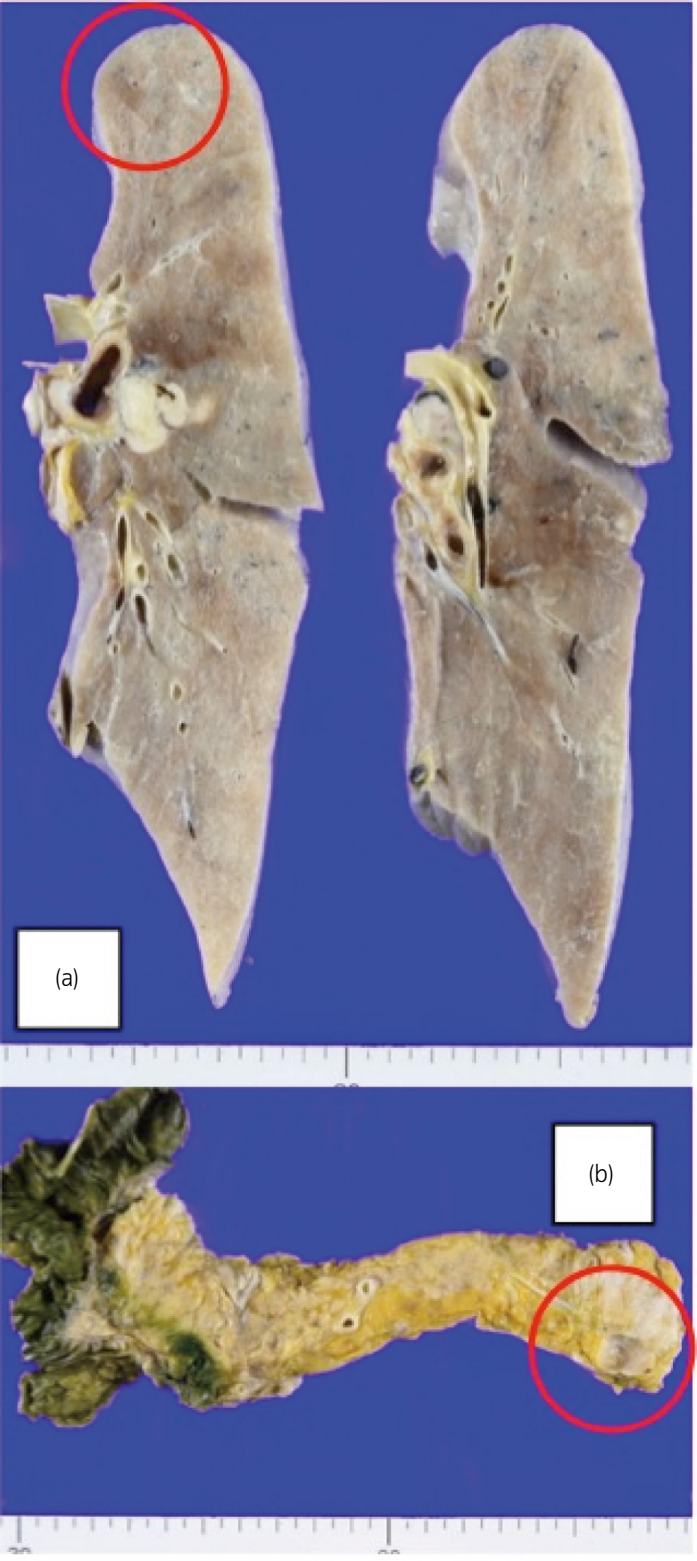
(a) Metastasis observed in the lungs (circled). (b) Metastasis observed in the distal pancreas (circled). [Colour figure can be viewed at wileyonlinelibrary.com]

Following consent from her family members, a post‐mortem examination was performed. Signet‐ring cell metastases were confirmed in the lungs, abdominal lymph nodes, retroperitoneum, distal pancreas, and much of the vertebral bones (Fig. 3a,b). No evidence of gastrointestinal lesions was observed. On immunohistochemistry, the SRCC cells stained positive for CK20 and negative for uroplakin II, CK7, and GATA3.

## Discussion

Upper urinary tract carcinomas account for 5–10% of all urinary tract malignancies, and histological variances are observed in roughly quarter of the cases.^1^ Of these variances, SRCC is extremely rare, with only a number of reported cases. The vast majority of urinary SRCCs are of lower urinary tract origin or secondary tumors. To the best of our knowledge, since first reported by Ekfors and Nurmi in 1988, fewer than 20 cases have been reported in the English literature.^1^, ^2^ Our case adds an additional significance, in its opportunity for an autopsy to thoroughly rule out other primary origins of this rare tumor.

There are three proposed pathogenesis of SRCC originating from the urinary tract. The first suggests that SRCC arises from metaplastic transformation of the urothelium or within areas of cystitis cystica. The second theory suggests that the tumor originates from normal, isolated signet‐ring cells that scatter along the urothelium. The final theory proposes that these cells arise directly from totipotent urothelial cells, without passing through metaplasia.^3^, ^4^, ^5^

The most common primary site for SRCC is the gastrointestinal tract.^1^, ^4^ SRCC in the urinary tract secondary to gastric SRCC has been frequently reported, and thus distinguishing the true origin of this tumor poses a clinical challenge.^3^, ^4^ Although we were unable to perform gastrointestinal endoscopy or colonoscopy following surgery to rule out the possibility of a gastrointestinal origin, we were able to definitively confirm this from her autopsy results.

Several attempts have been made to distinguish between primary and secondary SRCC of the urinary tract. Immunohistological analyses of these tumors have reported positive staining for markers such as CK7, CK20, CEA, epithelial membrane antigen, CDX2, villin, and E‐cadherin.^1^, ^3^, ^6^, ^7^ A negative CK7 and positive CK 20 profile was seen in this patient is a common feature in colonic adenocarcinoma,^8^ though this possibility was ruled out during the autopsy. Considerable overlap seems to exist between urinary and gastrointestinal SRCCs, which makes it difficult to differentiate the tumors at a histological level. Therefore, gastrointestinal endoscopy and colonoscopy to rule out the possibility of a gastrointestinal origin appear to be good clinical practice.^1^, ^3^

The clinical significance of serum tumor makers in SRCC has not been established. In advanced gastric cancer including gastric SRCC, CEA, and CA19‐9 levels are known to often increase, both of which were normal in our patient.^9^ Interestingly, Ye has also reported a case of upper urinary tract SRCC where these markers were not elevated.^1^ Although intriguing, the diagnostic relevance of this result is uncertain.

Unfortunately, there are currently no standardized management strategies for SRCC. Although there are sporadic reports on effective cases, this tumor is generally considered to be resistant to chemotherapy and radiotherapy, regardless of the organ of origin.^3^, ^7^, ^10^, ^11^ According to previous reports, Gemcitabine plus Carboplatin is often used as the first‐line therapy for bladder SRCC, and thus was our regimen of choice. Alternatively, fluorouracil (5‐FU) and platinum may also be considered, as it is the most frequented therapy for gastric SRCC.^10^ Additionally, the recent development in cancer treatment has opened up the potential use for immune checkpoint inhibitors, such as Pembrolizumab. Although data on the use of these drugs is very limited, reports on SRCCs from other organs suggest these tumors may possess a potentially favorable biomolecular profile for immunotherapy.^12^ Further exploration of immunotherapy in urinary SRCC patients is an anticipated area of research.

## Conclusion

We report a case of primary upper urinary SRCC. To the best of our knowledge, this is the first such a case with a post‐mortem examination, which allowed for an extensive confirmation of its primary origin.

## Author contributions

**Heisuke Iijima:** Conceptualization; writing – original draft. **Hiroaki Murata:** Validation. **Shinnosuke Oishi:** Validation. **Yusuke Kubota:** Validation. **Akihiko Sakamoto:** Validation. **Kuniaki Tanabe:** Validation. **Kazutaka Sugiyama:** Validation. **Akihiko Matsumoto:** Conceptualization; supervision; validation. **Ken Kuriki:** Supervision; validation. **Haruki Kume:** Validation.

## Conflict of interest

The authors declare no conflict of interest.

## Approval of the research protocol by an Institutional Reviewer Board

Not applicable.

## Informed consent

Not applicable (consent for autopsy and data publication taken from family members of the patient).

## Registry and the Registration No. of the study/trial

Not applicable.
